# Assessment of the health related quality of life in children with asthma in a tertiary hospital in North Central, Nigeria

**DOI:** 10.11604/pamj.2022.41.58.30171

**Published:** 2022-01-20

**Authors:** Helen Oluwadamilola Akhiwu, Nantok Dami, Stephen Oguche

**Affiliations:** 1 Department of Paediatrics, Jos University Teaching Hospital, Plateau State, Nigeria,; 2 Community Medicine Department, University of Jos, Plateau State, Nigeria

**Keywords:** Asthma, quality of life, children

## Abstract

**Introduction:**

asthma is a chronic inflammatory disorder of the airways with over 339 million people affected worldwide. Asthma can impair the quality of life (QoL) in its various bio-psycho-social domains causing poor concentration, poor school performance and impaired daily activities. This study assessed the QoL in asthmatic children aged 7- 17 years.

**Methods:**

a descriptive cross-sectional study of 46 children with asthma. Relevant bio-data and medical history were documented and the QoL assessment was carried out using the paediatric asthma quality of life questionnaire. Asthma severity and asthma control were defined based on the global initiative for asthma protocol. The data was analysed with IBM SPSS version 22.

**Results:**

the mean age was 12.4 ± 3.3 years, with a male to female ratio of 1: 1.9. About 61% of the study population were moderately impaired in their QoL and 41.3% had uncontrolled asthma. The mean QoL score was 5.80 with the activity domain and the emotion domain having the lowest mean scores (5.78 ± 1.0 and 5.91 ± 1.2 respectively). There was a significant association between QoL and age, asthma severity, asthma control and social class (p< 0.05) but not Gender. The logistic regression did not identify any of these factors as being predictors of QoL in the children. **Conclusion:** the study participants had moderately impaired QoL with the 7-10 year-olds more severely affected. The activity and emotion domains are more impaired. Therefore in addition to providing medical intervention, the treatment of children with asthma should include psychological support and counselling.

## Introduction

Asthma is a chronic inflammatory disorder of the airways [1]. According to the global initiative for asthma report of 2018 [2], it has been estimated that over 339 million people suffer from asthma world-wide. The disease also causes a high global burden of death and disability, with around 1000 people dying each day from the disease. Globally, asthma is ranked 16^th^ among the leading causes of years lived with disability and 28^th^among the leading causes of burden of disease [2]. The incidence of the disease the world over and even in children is on the rise [2]. It is therefore a global health problem for which appropriate actions for its control must be planned.

The quality of life (QoL) of any individual is affected by the environment as well as in response to certain diseases [3], therefore, it is important to evaluate the quality of life in patients with chronic diseases such as asthma, since the disease can impair the quality of life in its various bio-psycho-social domains. It also causes poor concentration; poor school performance as well as impairing the daily activities of the affected population [4,5]. The condition affects not only the individuals with the disease, but also their caregivers by altering the family dynamics [6-8].

The International Study of Asthma and Allergies (ISAAC) reported that asthma prevalence among children is increasing and Africa has contributed most to the burden of disease through its effects on quality of life [9]. Despite effective asthma medications available for symptom control, it has been reported globally that current treatments have not significantly reduced morbidity or mortality of the disease [10]. With this limitation therefore, there is an increasing need to use quality of life measures to evaluate the impact of asthma and its management on the daily lives and function of affected people and to identify the specific aspects of life with greater associated morbidity to enable effective management of the disease [11-13].

The measurement of health-related quality of life is also based on the growing recognition that health care should not focus only on the patient´s survival but also on the quality of life [14]. The information obtained from quality of life studies can help to guide clinical management as well as formulation of clinical guidelines and the equitable allocation of resources. There is however, very few quality of life studies carried out in Africa [15]. This study therefore aims to determine the quality of life of children with asthma and identify the specific domains affected to enable for targeted intervention.

## Methods

The study was a descriptive cross-sectional study of children aged 7-17 years attending the paediatrics respiratory clinic of a teaching hospital who presented with paediatric asthma and met the diagnostic criteria for asthma according to the 2017 GINA guidelines for the diagnosis of asthma in children [12].

Patients with underlying cardiac conditions or other respiratory morbidities (pulmonary tuberculosis, lung abscess etc.) were excluded from the study. The sample size was determined using the formula [15]:


$$n=\frac{n_{0}}{1+\frac{\left(n_{0}-1\right)}{N}}$$

Where


$$n_{0}=\frac{Z^{2}P\left(1-P\right)}{d^{2}}$$

n = the minimum sample size; Z = the standard normal deviate at 95% confidence level (1.96); d = maximum tolerable sample error (0.05); p = the proportion of the target population estimated to have asthma (using 0.10) [16] and N = number of children with asthma seen at clinic in 2017 (60 patients).

Thus,


$$n_{0}=\frac{{\left(1.96\right)}^{2}\times 0.10\left(1.0-0.10\right)}{\left(0.05\right)}=138.3$$

Therefore


$$n=\frac{138.3}{1+\frac{\left(138.3-1\right)}{60}}=\frac{138.3}{3.29}=42\text{ patients}$$

Eventually 46 patients were recruited for the study. Ethical approval was obtained from the institutional Ethics Committee (ethical approval number JUTH/DCS/ADM/127/XXIX/1643). While the parents or guardian of each patient signed an informed consent after the study had been explained to them. In addition, each patient received a patient information sheet on asthma. All patients provided child assent before the questionnaire was administered to them. Participants were allowed to opt out of the study at any time without loss of any benefits of the study or hospital care. The sampling technique was simple random sampling by balloting. An average of 10 patients with asthma are seen each week at the clinic. The list of the patients registered for the clinic was retrieved at the beginning of the clinic. These patients were then assigned a number from 1-n after which number 1-n were written on pieces of paper and rolled up then five numbers were selected by balloting. These made up the five patients recruited each clinic day for the study. This process was continued each week until the desired sample size was recruited.

Demographic data and medical history including age, sex, and socio-economic status and history of medication use were obtained and documented. For the purpose of this study each child was classified into a social class based on the criteria and computation of Oyedeji [17] whereby the occupational class and the education class of the parents are used to determine the social class of the child. The social classes are grouped from one to five where class 1 is the highest/upper social class while class 5 is the lowest social class. Quality of life assessment for all the participants was carried out using the generic interviewer-administered Paediatric Asthma Quality of Life Questionnaire (PAQLQ) [18]. This questionnaire was obtained and used with the permission from the authors. The PAQLQ 23 questions assess the child´s physical activity (5), emotions (8) and symptoms (10). Mean QoL scores from each domain and overall scores was calculated based on a seven-point scale. A QoL score of 7 was reported as best with no impairment; score of one as least and severest impairment; score 4 as mid-point in the range from 2-6 of moderate degree impairment.

Asthma severity was defined based on the Global Initiative for Asthma (GINA) protocol, that defines four categories; mild intermittent, mild persistent, moderate persistent and severe persistent. Asthma control was also defined based on GINA classification. The three categories being: controlled, partly controlled and uncontrolled. A child was considered either well controlled or not according to the guidelines [19].

**Data analysis:** the data obtained from the study was analysed using IBM SPSS statistics for windows version 22.0 (IBM Corp., Armonk, N.Y., USA). Initially, a univariate analysis of the socio-demographic characteristics (age, sex, and social class etc.) asthma severity and asthma control of the study participants was carried out and basic summary statistics produced for each variable. Quantitative variables were described using means and standard deviation. Qualitative variables were presented using frequency tables. The dependent variable is the quality of life while age, sex, asthma control, disease severity and socioeconomic characteristics were the independent variables.

The children were grouped into 3 age groups; 7-10, 11-14 and 15-17. The mean quality of life scores of each domain as well as the overall quality of life scores of each age group were calculated and presented as mean scores and standard deviation. To identify the factors that were associated with the quality of life, age, sex, asthma control, asthma severity and social economic class were tested. Analysis of variance (ANOVA) was used to identify the relationship between the different age groups and the QoL when a significant association was found; Bonferroni´s correction was used to identify which particular age group made the significant contribution that was identified. Chi-squared test then was used to determine the association between the quality of life and sex, social class, asthma control and asthma severity.

Logistic regression was used to determine the predictive variables. The outcome variable - quality of life- was converted into a binary outcome by grouping it into impaired and not impaired. Then sex, social class, asthma control and asthma severity were analyzed using logistic regression model to identify which of them was a predictor of the quality of life. A significance level of ≤ 0.05 was used for this study.

## Results

A total of 46 children with asthma were studied. The mean age was 12.4 ± 3.3 and the male to female ratio of the study population was 1: 1.9. Majority of the patients were in social class 2 with none of them in social class 4 and 5. About 61% of the study population were moderately impaired in their quality of life and almost half of them had uncontrolled asthma (Table 1). The most affected domain is the activity domain followed by the emotion domain in children with uncontrolled asthma while in the different categories of asthma severity the activity domain was the most impaired in all classes of severity (Table 2). There was a significant difference in the mean overall quality of life scores between the age groups studied and the age group 7-10 years was the most affected (Table 3). There was a significant association between quality of life and asthma severity, control and social class of the study population with p < 0.05. Gender however did not significantly affect the quality of life (Table 4). Neither asthma severity, asthma control, social class nor gender was a predictor of QoL in the children studied (Table 5).

**Table 1:** the socio-demographic characteristics, overall quality of life scores, asthma control and severity categories of the study population

Variable	Frequency (%)	Mean ± SD
**Age group (years)**		**12.4 ± 3.3**
7 - 10	12 (26.1)	
11 - 14	18 (39.1)	
15 - 17	16 (34.8)	
**Gender**		
Male	16 (34.8)	
Female	30 (65.2)	
**Grouped weight (kg)**		**39.1 ± 10.7**
21 - 30	13 (28.3)	
31 - 40	14 (30.4)	
41 - 50	8 (17.4)	
53 - 60	11(23.9)	
**Grouped height (cm)**		**148.3 ± 12.8**
131 - 140	18 (39.1)	
141 - 150	5 (10.9)	
151 - 160	13 (28.3)	
161 - 170	10 (21.7)	
**Social class**		
One	9 (19.6)	
Two	25(54.3)	
Three	12 (26.1)	
Four	0 (0)	
Five	0 (0)	
**Overall QOL scores**		
No impairment	18 (39.1)	
Moderate impairment	28 (60.9)	
Severe impairment	0 (0)	
**Asthma control**		
Well controlled	27 (58.7)	
Partially controlled	18 (39.1)	
Poorly controlled	1 (2.2)	
**Asthma control (binary)**		
Controlled	27 (58.7)	
Not controlled	19 (41.3)	
**Asthma severity**		
Intermittent	28 (60.9)	
Mild persistent	13 (28.3)	
Moderate persistent	5 (10.9)	
Severe persistent	0 (0)	

No impairment: scores of 7; moderate impairment: scores of 2-6; Kg: kilogramme; cm: centimetres

**Table 2:** quality of life scores for each domain in the different categories of asthma control and severity

	Variables	N	Mean	Std. deviation	Std. error mean
**Quality of life domains**	**Asthma control**				
QoL symptom	Controlled	27	6.66	0.48	0.09
	Not controlled	19	4.89	0.46	0.11
QoL activity	Controlled	27	6.67	0.48	0.09
	Not controlled	19	4.53	0.61	0.14
QoL emotion	Controlled	27	6.85	0.36	0.07
	Not controlled	19	4.58	0.61	0.14
**QoL symptom**	**Asthma severity**				
	Intermittent	28	6.61	0.56	0.11
	Mild persistent	13	5.00	0.00	.00
	Moderate persistent	5	4.60	0.89	0.40
QoL activity	Intermittent	28	6.61	0.57	0.11
	Mild persistent	13	4.69	0.48	0.13
	Moderate persistent	5	4.00	0.71	0.32
QoL emotion	Intermittent	28	6.79	0.49	0.09
	Mild persistent	13	4.77	0.44	0.12
	Moderate persistent	5	4.00	0.07	0.32

**Table 3:** distribution of the quality of life score for each age group, the analysis of variance test and the test of continuity test to determine which age group was significantly affected

Age groups							Mean values ± SD		
			**QoL total score**				**QoL symptom**	**QoL activity**	**QoL emotion**
For all ages			5.80 ± 1.2				5.93 ± 1.0	5.78 ± 1.0	5.91 ± 1.2
7 - 10			5.25 ± 1.14				5.50 ± 0.90	5.25 ± 1.14	5.25 ± 1.14
11 - 14			5.61 ± 1.29				5.78 ± 1.11	5.61 ± 1.29	5.61 ± 1.29
15 - 17			6.75 ± 0.68				6.44 ± 0.73	6.38 ± 0.89	6.44 ± 0.72
**ANOVA**									
**QoL total**									
		**Sum of squares**		**df**		**Mean square**		**F**	**p**
Between groups		10.774		2		5.387		4.590	0.016
Within groups		50.465		43		1.174			
Total		61.239		45					
**Bonferroni's correction to determine the age group that makes the significant difference in the overall quality of life scores**									
**Dependent variable: QoL total**									
**Bonferroni's**									
**(I) Age group**	**(J) Age group**		**Mean difference (I-J)**		**Std. error**		**p**	**95% confidence interval**	
								**Lower bound**	**Upper bound**
7 - 10	11 - 14		-0.36111		0.40373		1.000	-1.3669	0.6447
	15 - 17		-1.18750*		0.41370		0.019	-2.2181	-0.1569
11 - 14	7 - 10		0.36111		0.40373		1.000	-0.6447	1.3669
	15 - 17		-0.82639		0.37222		0.095	-1.7537	0.1009
15 - 17	7 - 10		1.18750*		0.41370		0.019	0.1569	2.2181
	11 - 14		0.82639		0.37222		0.095	-0.1009	1.7537

* : the mean difference is significant at the 0.05 level

**Table 4:** test of association between the QoL and asthma severity, control, socio-economic class and gender to determine the factors associated with quality of life

Variable	QOL scores					Mean QoL	Chi squared	df	P-value
	**3.0**	**4.0**	**5.0**	**6.0**	**7.0**				
**Asthma control**							**35.71**	**1**	<0.001*
Controlled	0	0	0	9	18	6.67			
Not controlled	1	6	12	0	0	4.57			
**Asthma severity**							**33.91**	**1**	<0.001*
Intermittent	0	0	1	9	18	6.61			
Mild persistent	0	3	10	0	0	4.77			
Moderate persistent	1	3	1	0	0	4.00			
**Social class**							**10.93**	**1**	0.001*
One	0	3	5	0	1				
Two	1	3	7	4	10				
Three	0	0	0	5	7				
**Gender**							**2.426**	**1**	**0.119**
Male	0	2	8	3	3				
Female	1	4	4	6	15				

None of the patients was in social class 4 and 5; *P value significant at £ 0.05

**Table 5:** logistic regression to identify which factors were the predictors of quality of life in the study population

Variable	P value	Odds ratio	95% confidence interval	
			Lower bound	Upper bound
Asthma severity	0.459	0.179	0.002	16.934
Asthma control	0.310	0.072	0.000	11.520
Social class	0.136	0.283	0.054	1.489
Gender	0.332	0.383	0.055	2.661

## Discussion

The mean overall quality of life score of the study population was 5.8. In addition, of the three domains that made up the overall scores, the activity and emotion domains were more impaired than the symptom domain. A mean quality of life of 5.8 in the study population means that on the average, children with asthma are moderately impaired in their quality of life. So the disease does affect the patient´s life as perceived by the patients. This value of 5.8 is higher than what has been documented in North Africa in a study in Egypt [20], where the overall quality of life was 4.7, the study from Poland [21] also reported a score of 4.4. The report from a study in India [22], which is also a developing country like ours, documented an overall quality of life score of 4.6. While a report from Bosnia and Herzegovina documented a mean QoL scores of 5.95 which is similar to this study despite the fact that it is an upper middle income country located in Europe [23] compared to Nigeria which is a lower middle income country located in Africa [24]. The study [15] in Abuja, Central Nigeria found a quality of life score of 4.9, which is lower than the value observed in this study.

Despite the fact that all the above mentioned studies had used the same quality of life assessment tool, the studies identified various degrees of impairment in the quality of life of the children studied. This shows that there must be a number of factors that determine the quality of life of children with asthma which is not necessarily just their environment or the affluence of the nation. It also buttresses the fact that the quality of life values varies from community to community and hence the need for local studies to be able to determine how to manage these patients better. Another reason for the relatively high quality of life observed in this study may be that though majority of the patients in this study were impaired in their quality of life, in more than half of them asthma was controlled thereby making the impairment in their quality of life not so severe. This study also observed that the physical activity and the emotion domains were the most affected domains, with the symptom domain being least affected. Meaning the children felt more impaired in their daily physical activities and emotionally. El-Gilany *et al*. [20] reported that the activity and symptom domains were more affected in their study while the emotion domain was the least impaired. While Ahmed *et al*. [15] in their study reported that that the physical activity domain was the least impaired. They had more impairment in the symptom and emotion domain. The study by Jovic *et al*. [10] found the children to be more impaired in the emotion domain than in the activities and symptom domains.

The exact reason for these varying presentations is not known but it might be that the disease that is causing a limitation in their daily activities making them unable to play with their peers may also have a negative impact on the children emotionally. It is therefore important to note that with varying affectation of the three domains in different studies, the need for local studies to help with patient management cannot be over emphasized. It is also important to note that in addition to providing medical intervention, the treatment of children with asthma should include psychological support and counselling. This reasoning has been supported by other authors [10,25]. The age, gender, social class, asthma control and asthma severity were tested to determine the factors that were associated with the QoL. It was observed that age, asthma control, asthma severity and social class were significantly associated with the quality of life in children with asthma while gender was not significantly associated with the quality of life of children with asthma. When the overall QoL score was stratified by age it was observed that the quality of life was lower in the younger children when compared with the older children. The mean difference in the quality of life scores were then compared between the groups and it was observed that this difference was statistically significant and the 7-10 years´ age group contributed the significance.

This may be because the younger aged children are new to their diagnosis and are still trying to understand its ramifications on all aspects of their life while the older age groups have had a longer time to adapt to their diagnosis and the consequences. This finding is similar to what was reported by Ahmed *et al*. [15] who also documented that the quality of life was poorer in the lower age groups although the difference was not significant in that study. El- Gilany *et al*. [20] in Egypt also documented mean quality of life scores that were higher in the lower aged children though this difference was also not statistically significant. The studies by Moreira *et al*. [26], Blaakman *et al*. [27] and Mosnaim *et al*. [28] on the other hand however, documented that QoL worsens with age because the older children tend to be less adherent to their medications and treatment regimes.

Asthma control was found to be significantly associated with the QoL of children with asthma. It has been reported by other authors [11,29] as well, that poor asthma control is associated with frequent symptoms, limitation of activities and worry about asthma attacks hence worsening the quality of life of the children involved [11,29]. This should not be too surprising because, subjects with well controlled asthma will require less use of medications and have less frequent need for hospital visit for emergency care that burdens the family finances and impart on quality of life. This study therefore brings to the fore that asthma control is important if we are to improve the quality of life of these children. This study also found that asthma severity was significantly associated with QoL. With the more severe the asthma was, the poorer the quality of life. This is similar to the findings of a number of previous studies [30-32], who had also observed this relationship where it was reported that the more severe asthma is, the poorer the quality of life in children under 18 years especially if the child had moderate to severe persistent asthma. This is however, not always the case as some other studies [33,34] could not find an association between asthma severity and child´s QoL.

Neither asthma severity, asthma control, social class nor gender predicts QoL in the children studied. However, further analysis showed that for children with controlled asthma the QoL scores in the symptom and activity domains were the ones most affected while they were least affected in the emotion domain. While in children with uncontrolled asthma the activity domain and emotion domains were the most affected while the symptom domain was least affected. Children with controlled asthma, were least affected in their emotion domain and this could be explained to be as a result of the fact that more control meant they had fewer exacerbations, fewer individuals are aware of their disease and this may not affect their relationship with their peers hence having a lower emotional toll on them. The findings in those children with uncontrolled asthma who usually develop acute exacerbations at any time and more frequently are not entirely surprising. This is because poor control and more frequent exacerbations will indeed lead to limitation of activity and this in turn would affect these children emotionally as they cannot mingle well with their peers and may even end up being stigmatized. The children with intermittent asthma were more impaired in the physical activity and symptom domain and least affected emotionally while those with mild persistent were most impaired in their activity domain and those with moderate persistent asthma were equally impaired in their activity and emotion domains and least impaired in their symptom domains.

Ahmed *et al*. [15] in their study documented that asthma severity and poor control were independent predictors of quality of life in children with asthma. The authors were of the opinion that the fact that some of the children miss school, are not able to participate in certain physical activities or work due to their asthma can lead to impairment that may be physical, psychological or social and these factors tend to be more pronounced when the sufferer has severe or poorly controlled asthma. Al-Gewely *et al*. [35] also documented that the emotion domain was more affected in children with uncontrolled asthma than the other categories of asthma control. Another study from the Arab world revealed that asthma had a significant adverse effect on the quality of life of children as indicated by the high prevalence of behavioural and emotional difficulties among them, in addition to increased frequency of school absenteeism and deteriorating academic performances [36]. Poor asthma control was also associated with clinically significant levels of behavioural problems, in children with asthma studied in the United States [37]. While some other studies [38,39] have reported that anxiety and depression are prevalent in those children with poor asthma control as well as behavioural disturbances being more evident among children with severe asthma. These findings therefore emphasize that poor asthma control and increased levels of asthma severity have effects not only on the overall quality of life but also specifically the emotional domains of the children with the disease.

**The limitation of this study:** the sample size though calculated based on the number of patients seen is small and the findings may not be generalizable to the entire population. A multicentre study with a larger population would be recommended to further support the findings of this study.

## Conclusion

The study participants were moderately impaired in their quality of life with the children aged 7-10 years being most severely affected. The physical activity and emotion domains are more impaired. Age, asthma severity, asthma control and socioeconomic class were the factors significantly associated with the quality of life in these children.

**Recommendations:** it is our recommendation that assessment of the quality of life of children with asthma should be included as part of their standard of care so that patient management can be specifically tailored to each patient. In addition to providing medical intervention, the treatment of children with asthma should include psychological support and counselling at the time of diagnosis to help them deal with their disease. They should also be provided with counselling whenever the managing physician judges that they are in need of it.

**Source of funding statement:** the research reported in this publication was supported by the Fogarty International Centre (FIC) of the National Institutes of Health and also the Office of the Director, National Institutes of Health (NIH), National Institute of Nursing Research (NINR) and the National Institutes of Neurological Disorders and Stroke (NINDS) under award number D43TW010130. The content is solely the responsibility of the authors and does not necessarily represent the official views of the National Institutes of Health.

### What is known about this topic

- *The incidence of asthma in children is on the rise;*
- *Despite effective asthma medications available for symptom control, the current treatments have not significantly reduced morbidity or mortality of the disease;*
- *There is an increasing need to use quality of life measures to evaluate the impact of asthma and its management on the daily lives and function of affected children and to identify the specific aspects of life with greater associated morbidity to enable effective management of the disease*.

### What this study adds

- *This study showed that children with asthma had an impaired quality of life;*
- *The study was able to identify that the emotion and activity domains of the quality of life were more impaired in children with asthma, therefore in addition to providing medical intervention, the treatment of children with asthma should include psychological support and counselling;*
- *This study also demonstrated that age, social class, asthma severity and control and not gender were the factors significantly associated with the quality of life in children with asthma*.
